# Editorial: Primary membranous nephropathy

**DOI:** 10.3389/fimmu.2022.1102036

**Published:** 2022-12-06

**Authors:** Maria Infantino

**Affiliations:** Laboratory of Immunology and Allergology, S. Giovanni di Dio Hospital, Florence, Italy

**Keywords:** idiopathic membranous nephropathy, anti-phospholipase A2 receptor (PLA2R) autoantibodies, biomarker, nephrotic syndrome, antibodies

Membranous nephropathy (MN) is the main cause of nephrotic syndrome in adults. MN can be either idiopathic (iMN) or secondary (sMN) to various clinical conditions, including systemic autoimmune diseases, infections, neoplasia, drug use, and heavy metal poisoning. The pathogenesis is complex, characterized by the formation of immune deposits, complement-mediated proteinuria, and risk of renal failure. The immune complexes comprise immunoglobulin G and some important antigens, such as phospholipase A2 receptor (PLA2R), thrombospondin domain-containing 7A (THSD7A), and complement components. In 2009, Beck et al. (1) detected the co-location of PLA2R and IgG antibody in renal pathological sections of iMN patients, and detected the existence of PLA2R autoantibodies in serum samples, thus identifying PLA2R as the main autoantigen of iMN. Subsequently, other target antigens of iMN, such as THSD7A, semaphorin 3b (Sema3b), neural epidermal growth factor-like 1(NELL1), protocadherin 7 (PCDH7), and high-temperature recombinant protein A1 (HTRA1) were identified. The discovery of these autoantigens deepened our understanding of the pathogenesis of MN and improved its diagnostics. The prevalence of anti-PLA2R antibodies in iMN patients ranges between 30 and 89% depending mainly on ethnic population and detection method (western blot, indirect immunofluorescence assay and enzyme linked immunosorbent assay), all displaying high diagnostic specificity and high concordance. Anti-PLA2R antibodies not only have an important role in diagnosis (“the liquid biopsy”) but are also indicators to evaluate the disease state of iMN patients, which can be used to guide clinical treatment by monitoring patients according to the KDIGO guidelines. Hence this marker is important for treatment options and to predict a decrease in proteinuria over several months in patients with MN.

In their research article Dong et al. demonstrated in a large Chinese cohort that hypercholesterolemia at baseline is an independent risk factor for persistent proteinuria in iMN, with a good correlation with glomerular and tubular lesions, glomerular PLA2R deposit, and serum anti-PLA2R titers. They also demonstrated that dyslipidemia may be a potential therapeutic target in iMN. They concluded therefore that hypercholesterolemia may be considered a potentially useful biomarker for disease severity in the outcome of iMN.

In a multicentre study Liu et al. investigated the association between serum C4 level at renal biopsy and kidney disease progression in 328 patients with iMN showing that a higher level of C4 was associated with a higher risk of renal function progression events or end-stage renal disease (ESRD). C4 at renal biopsy has been proposed as an independent predictor for kidney disease progression regardless of other confounders.

Zhang et al. showed that the level of IL-35 in patients with iMN in the remission stage was higher than during active disease and trended upward as treatment progressed. The baseline IL-35 could predict the remission time of iMN patients, including those patients with negative aPLA2R. The level of IL-35 was related to the number of iTR35 cells, reflecting the immune status of patients, and different IL-35 levels may reflect different choices for the treatment of iMN.

So et al. highlighted in their review article the role of complement cascade activation in primary MN, in particular with the mannose-binding lectin (MBL) and alternative pathways implicated. Activation of the complement system has emerged as a key contributor not only in diagnosing MN but also in prognosis and treatment strategy.

Liu et al. investigated traditional and new biomarkers in iMN. PLA2R is the most well-established target antigen in the disease but proteins, metabolites, noncoding RNAs (ncRNAs), and immune cells have recently been found as novel antigens. Although these new markers have not been used in clinical settings and need to be verified, they may guide the therapeutic approach for MN and have a great significance in the diagnosis, progression, prognosis, and treatment response of disease.

## Author contributions

The author confirms being the sole contributor of this work and has approved it for publication.
